# Mapping of Recognition Sites of Monoclonal Antibodies Responsible for the Inhibition of Pneumolysin Functional Activity

**DOI:** 10.3390/biom10071009

**Published:** 2020-07-08

**Authors:** Indre Kucinskaite-Kodze, Martynas Simanavicius, Justas Dapkunas, Milda Pleckaityte, Aurelija Zvirbliene

**Affiliations:** Institute of Biotechnology, Life Sciences Center, Vilnius University, Sauletekio av. 7, LT-10257 Vilnius, Lithuania; martynas.simanavicius@bti.vu.lt (M.S.); justas.dapkunas@bti.vu.lt (J.D.); milda.pleckaityte@bti.vu.lt (M.P.); aurelija.zvirbliene@bti.vu.lt (A.Z.)

**Keywords:** neutralising antibodies, pneumolysin, pore-forming toxins, epitope mapping

## Abstract

The pathogenicity of many bacteria, including *Streptococcus pneumoniae*, depends on pore-forming toxins (PFTs) that cause host cell lysis by forming large pores in cholesterol-containing cell membranes. Therefore, PFTs-neutralising antibodies may provide useful tools for reducing *S. pneumoniae* pathogenic effects. This study aimed at the development and characterisation of monoclonal antibodies (MAbs) with neutralising activity to *S. pneumoniae* PFT pneumolysin (PLY). Five out of 10 produced MAbs were able to neutralise the cytolytic activity of PLY on a lung epithelial cell line. Epitope mapping with a series of recombinant overlapping PLY fragments revealed that neutralising MAbs are directed against PLY loops L1 and L3 within domain 4. The epitopes of MAbs 3A9, 6E5 and 12F11 located at L1 loop (aa 454–471) were crucial for PLY binding to the immobilised cholesterol. In contrast, the MAb 12D10 recognising L3 (aa 403–423) and the MAb 3F3 against the conformational epitope did not interfere with PLY-cholesterol interaction. Due to conformation-dependent binding, the approach to use overlapping peptides for fine epitope mapping of the neutralising MAbs was unsuccessful. Therefore, the epitopes recognised by the MAbs were analysed using computational methods. This study provides new data on PLY sites involved in functional activity.

## 1. Introduction

*Streptococcus pneumoniae* (pneumococcus) is the most common cause of bacterial otitis media, pneumonia, meningitis, sepsis and other severe illnesses [1]. This bacterium is the main etiological agent of severe pneumonia, causing up to 45% of pneumonia cases [2]. High morbidity and mortality related to pneumococcal infections demonstrate the need for novel treatment strategies. The currently available pneumococcal vaccines based on polysaccharide capsules can protect from about a quarter of known *S. pneumoniae* serotypes [3]. However, they do not protect from colonisation or infection by nonencapsulated pathogenic pneumococci [3,4]. Pneumolysin (PLY), a pore-forming toxin (PFT) produced by pneumococcus, is a major protein virulence factor and a potential candidate for developing protein-based vaccines [5]. It is well-recognised that PLY plays a significant role in severe outcomes of pneumococcal disease, in particular in the pathogenesis of lung and myocardial dysfunction [6]. Development of pneumococcal disease leads to the dysfunction of the endothelial barrier, increasing its permeability and formation of pulmonary edema in the lungs. The edema formation correlates with the presence of PLY [7]. The pathogenic effects of PLY were also confirmed in animal models of pneumonia [8,9]. Therefore, strategies for neutralisation of the toxic activity of PLY might provide a tool for reducing *S. pneumoniae* pathogenicity.

PLY belongs to the cholesterol-dependent cytolysin (CDC) family [10]. Oligomers of these toxins form large transmembrane pores consisting of 30–50 monomers in the cholesterol-containing cell membranes [11,12]. The virulence of CDCs is mainly related to barrier dysfunction caused by cell attack. The crystallographic analysis of PLY protomers revealed characteristic structure consisting of four functional domains [13,14]. PLY monomer, like other CDCs, interacts with cholesterol-rich cell membrane through its domain 4 (D4) [13]. Prepore-forming PLY monomers assembled into oligomers on the cell membrane undergo critical structural changes in domain 3 (D3): alpha helical bundles (α-HB1 and α-HB2) transform into β hairpins (TMH1 and TMH2) and perforate target membrane [15].

D4 is responsible for docking and anchoring of CDC to cholesterol in the cell membrane. The tip of D4 consists of four loops. The undecapeptide (UDP) loop is highly conserved among CDCs and forms an interaction site with membrane [16]. Moreover, the UDP is the element that couples membrane binding and allosteric changes in D3 leading to pore formation [17]. The cholesterol-recognition motive (CRM) of PLY composed of T459–L460 pair located in the loop 1 (L1) [18]. Modulation of CDC binding properties is realised by the structure of loop 3 (L3) that allows the discrimination of the lipid environment of the membrane [18].

Besides pore formation, PLY has other ways of its pathogenic action on host cells. Recent data suggest that PLY at sublytic doses may allow pneumococci to invade alveolar macrophages and monocyte-derived dendritic cells by inhibiting proinflammatory cytokine responses, thus avoiding cell resistance to pneumococci [19]. The cytoskeleton rearrangement and proinflammatory responses could also be induced at sublytic doses of PLY [7,20,21,22,23].

Antibodies can be used directly for the elimination of CDC cytolytic or other harmful activity by blocking CDC binding to a cellular receptor or by interfering with CDC oligomerisation. The neutralising monoclonal antibodies (MAbs) were developed against several PFTs, including streptolysin O [24], listeriolysin O [25,26], vaginolysin [27] and PLY [28]. The neutralising MAb PLY-5 recognising the undecapeptide conserved among all CDCs involved in the interaction with cell membrane was identified [29]. The previously developed CDC-specific monoclonal and recombinant antibodies were used for identification of CDC regions involved in cytolytic activity, and also for studying CDCs structure and conformational states [24,25,26,27,28,30,31].

In this study, we have employed a series of neutralising MAbs against PLY to investigate their potential to neutralise (inhibit) PLY pathogenic effects, such as cytolytic activity and binding to a cellular receptor. By combining experimental and computational approaches, we have investigated in detail the epitopes of the MAbs and identified a unique neutralising MAb directed to the cholesterol-binding loop of PLY and showing a broad specificity to several CDCs.

## 2. Materials and Methods

### 2.1. Recombinant Cytolysins

Recombinant N-terminal hexahistidine tag (His-Tag) containing cytolysins: *Streptococcus pneumoniae* pneumolysin (PLY), *Gardnerella spp.* vaginolysin (VLY), *Streptococcus intermedius* intermedilysin (ILY), *Clostridium perfringens* perfringolysin O (PFO), *Listeria monocytogenes* listeriolysin O (LLO), *Streptococcus pyogenes* streptolysin O (SLO) were expressed and purified as previously described in [27]. Production of *Lactobacillus iner* inerolysin (INY) was described in [32]. Briefly, the corresponding cytolysin-coding DNA lacking a putative signal sequence, except for PLY, was amplified from the respective bacterial isolates. The obtained PCR products were sequenced, cloned into pET28a(+) vector (Thermo Fisher Scientific, Waltham, MA, USA), and transformed into *E. coli* BL21 (DE3) strain (Merck KGaA, Darmstadt, Germany). Recombinant cytolysins were purified using Ni-chelate affinity chromatography columns (HisTrap HP, 17-5247-01, GE Healthcare Bio-Sciences AB, Uppsala, Sweden) according to the manufacturer’s recommendations.

### 2.2. Synthesis of Truncated PLY Fragments

Epitopes recognised by the MAbs were mapped using a set of truncated PLY fragments. For the epitope mapping, PLY was divided into eight overlapping truncated protein fragments (Table 1).

These fragments were synthesised in *E. coli*, as described previously [33]. Briefly, the DNAs containing the coding sequences of the PLY fragments were amplified by PCR. The primers (Metabion International AG, Planegg, Germany) used to generate PLY1–PLY8 fragments are listed in Table 2. The amplified DNA fragments were verified by sequencing, cloned the pET16b plasmid (Merck KGaA), and the resulting plasmid was transformed into *E. coli* Tuner (DE3) strain (Merck KGaA). After induction with isopropyl β-d-1-thiogalactopyranoside (IPTG), the synthesis of recombinant N-terminally hexahistidine tagged protein fragments was confirmed both by 12.5% polyacrylamide gel electrophoresis (SDS-PAGE) under reducing conditions and Western blot (WB) using anti-His-Tag MAb (MA1-21315, Thermo Fisher Scientific). The reactivity of the MAbs with the truncated PLY fragments was analysed by WB.

### 2.3. Development of Monoclonal Antibodies (MAbs)

MAbs were developed according to standard procedure that involves the immunisation of mice, cultivation of myeloma cells, cell hybridisation, hybridoma selection, cloning of hybridomas, evaluation of MAbs specificity to PLY and cross-reactivity to related proteins, determination of immunoglobulin classes and subclasses, MAb purification and cryopreservation of hybridomas.

Four eight-week-old BALB/c mice were immunised with 50 µg of recombinant PLY by subcutaneous injection three times every 28 days. For the first and the second immunisations, 50 μg of PLY was emulsified with complete (77140, Thermo Fisher Scientific) and incomplete (77145, Thermo Fisher Scientific) Freund’s adjuvants, respectively. After 56 days, mice were immunised with the same dose of antigen dissolved in phosphate-buffered saline (PBS). Blood samples were collected by tail bleeding before each and 28 days after the last immunisation, frozen, and stored at −20 °C until analysis. Mice for immunisation experiments were obtained from the breeding colony of the Centre for Innovative Medicine (Vilnius, Lithuania). Animal maintenance and experimental protocols were performed in accordance with FELASA guidelines and Lithuanian and European legislation (License No. LT-59-902, Permission No. 184 for breeding of mice, and Permission No. 209 for generation of polyclonal and monoclonal antibodies issued by the State Food and Veterinary Service, Vilnius, Lithuania).

Blood samples of the immunised mice were analysed by an indirect enzyme-linked immunosorbent assay (ELISA). A titre of specific antibodies to PLY was determined, and the mouse with the highest antibody titre was selected for hybridisation. Three days before hybridisation, the mouse was boosted with 50 μg of PLY dissolved in PBS.

Viable myeloma SP2/0 cells (CRL-1581, ATCC) and spleen cells isolated from the immunised mouse in ratio 1: 5 were fused with polyethylene glycol/dimethyl sulfoxide solution (PEG/DMSO, P7306, Merck KGaA). Fused cells were cultivated in Dulbecco’s modified Eagle’s growth medium (DMEM, F 0445, Merck) supplemented with hybridoma selection reagent hypoxanthine/aminopterin/thymidine (HAT, H0262, Merck KGaA), 2 mM L-glutamine, 200 µg/mL gentamicin, and 15% fetal bovine serum (FBS, S 0615, Merck KGaA,) in 96-well plates (92096, TPP, Trasadingen, Switzerland) at 37 °C and 5% CO_2_ for five days. After, growth medium was replaced with DMEM supplemented with hypoxanthine/thymidine (HT, H0137, Merck KGaA), 2 mM L-glutamine, 200 µg/mL gentamicin, and 15% FBS.

Ten days after fusion, cells were examined under a microscope. The growth medium of viable hybridomas was tested by an indirect ELISA for the presence of PLY-specific antibodies. Hybridomas producing MAbs to PLY were cloned by limiting dilution in DMEM containing 2 mM L-glutamine, 200 µg/mL gentamicin, and 15% FBS in 96-well plates (92096, TPP) at 37 °C and 5% CO_2_. Positive clones were selected by an indirect ELISA and propagated in 24-well plates (92024, TPP) for cryopreservation.

For all described experiments, affinity-purified MAbs were used. MAb purification from hybridoma growth medium was performed by affinity chromatography with protein A packed HiTrap Protein A HP columns (17-0402-01, GE Healthcare Bio-Sciences AB) according to the manufacturer’s recommendations and ÄKTA start chromatography system (GE Healthcare Bio-Sciences AB). NanoDrop UV-Vis spectrophotometer (NanoDrop™ 2000, ND-2000,Thermo Fisher Scientific) was used for MAb concentration measurements.

### 2.4. Enzyme-Linked Immunosorbent Assay (ELISA)

The reactivity of antibodies with PLY and other cytolysins was investigated by an indirect ELISA. Ninety-six-well micro test plates (10-121-0000, Nerbe plus, Winsen/Luhe, Germany) were coated with 50 µL of the corresponding antigen at 5 µg/mL concentration in a coating buffer (50 mM sodium carbonate, pH 9.5) for 16 h at 4 °C. The uncoated surface of the plates was blocked for 1 h at room temperature (RT) with 300 µL of 2% bovine serum albumin (BSA) in PBS. After blocking, the incubation with 100 µL of antibody samples was carried out for 1 h at RT. Hybridoma supernatants were used undiluted. For determination of the apparent dissociation constants (K_d_), the purified MAbs were diluted in PBS containing 0.1% of Tween-20 (PBST) in a range of concentration from 134 to 0.033 nM. After incubation, the plates were washed three times with PBST. The antibody-antigen complexes were detected by anti-mouse IgG conjugated with horse-radish peroxidase (HRP). The plates with 50 µL per well of the secondary antibody anti-mouse IgG-HRP (1721011, Bio-Rad, Hercules, CA, USA) diluted 1: 5000 in PBST were incubated for 1 h at RT. After incubation, plates were washed six times with PBST, and the enzymatic reaction was achieved by adding 100 µL of 3,3′,5,5′-tetramethylbenzidine (TMB, 01016-1, Clinical Science Products, Mansfield, MA, USA). It was stopped with 1 N H_2_SO_4_. Optical density (OD) was measured by a microplate spectrophotometer (Multiscan GO, 51119200, Thermo Fisher Scientific) and calculated as the difference between OD values at 450 and 620 nm (reference wavelength).

The apparent dissociation constant (K_d_) values of the MAbs were determined from their titration curves. The K_d_ value corresponds to MAb concentration (nM) at which the OD decreased by 50%.

Fine epitope mapping was performed using a set of N-biotinylated synthetic peptides spanning the respective PLY fragments (aa 196–220, aa 382–423 and aa 454–471) with linker (SGSG) (Pepscan, Lelystad, The Netherlands). Peptide sequences are presented in Table 3. For the epitope mapping, MaxiSorp plates (Nunc, 44-2404-21, Thermo Fisher Scientific) were coated with 50 µL of avidin (1405-69-2, Thermo Fisher Scientific) diluted with deionised water at 5 µg/mL final concentration. The plates were dried at 37 °C overnight. The plates were blocked with 300 µL/well of 2% BSA in PBST. After incubation, the plates were washed and 100 μL of biotinylated peptides at 10 μg/mL diluted in PBS were added to the wells. The plates were incubated for 1 h at RT. After washing, the plates were incubated with 100 µL of MAbs diluted until 5 µg/mL in PBST and incubated for further 1 h at RT. The steps of incubations with the secondary antibody and the substrate were performed as described above.

The inhibition of PLY interaction with cellular mannose receptor C type 1 (MRC-1) by the MAbs was evaluated by an indirect ELISA with some modifications. Briefly, 96-well plates MaxiSorp were coated with 50 µL/well of recombinant human MRC-1 (2534-MR, R&D Systems, Minneapolis, MN, USA) diluted to 2 µg/mL in a coating buffer (50 mM sodium carbonate, pH 9.5) for 16 h at 4 °C. The wells were blocked with 300 µL of 20% FBS in PBS for 2 h at RT. Then plates were washed three times with PBS containing 0.01% Tween-20. MAbs were diluted with PBS containing 0.01% Tween-20 at concentrations ranging 200 to 3 nM and preincubated in special tubes with low protein binding (0030108116, Eppendorf, Hamburg, Germany) with 72 nM of PLY for 30 min. One hundred µL/well of prepared MAb-PLY mix was loaded to the wells for 1 h at RT. After repeated washing procedure, the MAb-PLY complexes bound to MRC-1 were detected with HRP-conjugated anti-mouse IgG as described above.

The reactivity of MRC-1 with synthetic peptides was evaluated by a modified direct ELISA. The recombinant MRC-1 (2534-MR, R&D Systems) was immobilised to the wells of the MaxiSorp plate and blocked as described above. After washing, biotinylated peptides were diluted with PBS containing 0.01% Tween-20 at concentrations ranging from 1.25 to 20 µg/mL. One hundred µL/well of peptides were loaded to the wells and incubated for 2 h at RT. After incubation the plate was washed four times with PBS containing 0.01% Tween-20. Fifty µL/well of streptavidin-alkaline phosphatase (7105-04, SouthernBiotech) diluted 1: 2000 in PBS containing 0.01% Tween-20 were loaded to the wells for 30 min. After incubation, the plate was washed six times with PBS containing 0.01% Tween-20 and the enzymatic reaction was developed by adding 200 µL of p-nitrophenylphosphate (pNPP, P7998, Merck KGaA). The reaction was stopped with 3 M NaOH. The OD was measured by a microplate spectrophotometer (Multiscan GO) at 405 nm.

### 2.5. Western Blot (WB)

The reactivity of the MAbs to PLY and related cytolysins was evaluated by WB. Recombinant cytolysins at 1 µg/lane or 20 µL of bacterial lysate after boiling in a reducing sample buffer (39000, Thermo Fisher Scientific) were electrophoretically fractionated in 12.5% SDS-PAGE mini gel using SDS-Tris-Glycine buffer. Proteins from the SDS-PAGE were transferred to the polyvinylidene-difluoride (PVDF) membrane (T830.1, Carl Roth, Karlsruhe, Germany) under semidry conditions. The membranes were blocked with 2% milk powder (T145.3, Carl Roth) in PBS by incubating overnight at 4 °C. After blocking, the membranes were incubated for 1 h at RT with the purified MAbs (for 1 cm^2^ membrane sheet, 0.2 mL of the MAb at a concentration of 2 µg/mL were used). After washing in PBST, the membranes were incubated for 1 h with anti-mouse IgG-HRP (1721011, Bio-Rad) at RT. After rinsing the bands of proteins were developed with ready-to-use TMB solution (37574, Thermo Fisher Scientific). The reaction was stopped by immersing the membranes into water.

The reactivity of the MAbs with the wild-type PLY from cultured clinical *S. pneumoniae* isolates, was performed by WB as described above. The clinical isolate cultures 6477, 6840, 6958, 7123, 7144 were obtained from The National Public Health Laboratory (Vilnius, Lithuania) as lysates in PBS with 2% of sodium dodecyl sulphate (SDS).

### 2.6. Cholesterol Binding Test by Dot Blot

Cholesterol (Merck KGaA) dissolved in chloroform at a concentration of 1 µM of was spotted on a nitrocellulose membrane (10600002, GE Healthcare Bio-Sciences AB), 2 µL/spot. The membrane was dried for 30 min and blocked with 3% of milk powder (T145.3, Carl Roth) in PBS with 0.01% Tween-20 for 1 h at RT. The MAbs 3A9, 3F3, 6E5, 12D10 and 12F11 were diluted in PBS containing 0.01% Tween-20 at 72 nM concentration and preincubated with 36 nM of PLY in protein low-binding tubes (Protein LoBind Tubes, 0030108116, Eppendorf) for 30 min. MAb-PLY mixes were applied to the membranes for 1 h at RT. After washing, the membranes were incubated with the detection MAb 14E4 diluted in PBS containing 0.01% Tween-20 at 72 nM for 1 h at RT. After incubation, the membranes were washed and incubated with biotinylated goat antimouse IgG Fc antibodies (1033-08, SouthernBiotech, Birmingham, USA) diluted 1: 5000 in PBS containing 0.01% Tween-20 for 1 h at RT. Then the membranes were washed and incubated with streptavidin-alkaline phosphatase (7105-04, SouthernBiotech) diluted 1: 2000 in PBS containing 0.01% Tween-20 for 30 min at RT. After washing, the spots were developed with BCIP/NBT chromogen (34042, Thermo Fisher Scientific) for 30 min. The reaction was stopped by rinsing the membranes in water.

### 2.7. Determination of MAb Neutralising Activity

The cytolytic activities of recombinant cytolysins (PLY, ILY, VLY, PFO, LLO, INY and SLO) and their neutralisation by the MAbs were investigated using adherent human lung epithelial A549 cells (A549, CCL-185, ATCC). The cells were cultivated in 96-well plates (92096, TPP) in DMEM supplemented with 10% FBS, and propagated until cell confluence reached 70–90% at 37 °C and 5% CO_2_. Before the cytolysis and its neutralisation, the cells were rinsed twice with serum-free medium. 

For the measurement of cytolytic activity, each cytolysin at different concentrations ranging from 250 to 0.2 nM was diluted in serum-free medium and applied to the cells (100 µL per well). After incubation for 1 h at 37 °C and 5% CO_2_, cell viability was measured by adding 20 µL of MTS solution ([3-(4,5-dimethylthiazol-2-yl)-5-(3-carboxymethoxyphenyl)-2-(4-sulfophenyl)-2H-tetrazolium, inner salt (MTS) with phenazine methosulfate (PMS)) from CellTiter 96^®^ AQueous Non-Radioactive Cell Proliferation Assay (Promega, Madison, WI, USA) and incubated for 2 h. The OD was measured at 490 nm and 630 nm as a reference. The concentration of each cytolysin that lysed 50% of the cells was calculated from the dose-dependent curve and considered as 1 hemolytic unit (HU).

For the determination of the neutralising activity of the MAbs, the respective MAbs at serial twofold dilutions ranging from 200 to 6.25 nM were preincubated with 1 HU of the respective cytolysin for 30 min. The prepared mix was applied to rinsed cells (100 µL per well) and incubated for 1 h at 37 °C and 5% CO_2_. After incubation, cell viability was measured using MTS solution as described above. The concentration of the MAb, which inhibited 1 HU of cytolysin-induced lysis by 50% was considered as one neutralising unit (NU).

Serum-free medium with the analysed MAb only (without the cytoysin) was used as a control for full neutralisation (100% neutralisation), the cytolysin without any MAb was used as a control for full cytolysis (0% neutralisation).

### 2.8. Computional Analysis of PLY Epitopes Involved in Cytolytic Activity

The sequences of given cytolysins were aligned using the MAFFT software [34]. The available structures of cytolysins were downloaded from the Protein Data Bank (PDB) [35], and the D4 structure was extracted from these structures (Table 4). The D4 of INY was modelled using HHpred and Modeller from MPI Bioinformatics Toolkit [36,37], using the PDB structure 5CR8 as template. Protein structures and their solvent-accessible surfaces were compared using CAD-score [38] available in the Voronota package [39].

## 3. Results

### 3.1. Development of the MAbs Against PLY

**Figure 1:**
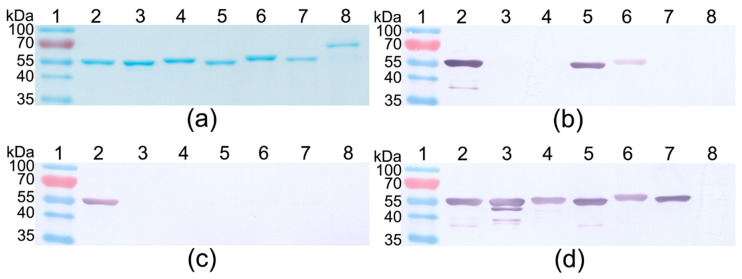
The reactivity of the MAbs with recombinant cytolysins PLY (lane 2), ILY (lane 3), VLY (lane 4), PFO (lane 5), LLO (lane 6), INY (lane 7) and SLO (lane 8). (**a**) SDS-PAGE. (**b**) WB with MAb 3A9. (**c**) WB with MAb 6E3. (**d**) WB with MAb 6E5. Lane 1, prestained molecular weight marker (PageRuler™ Prestained Protein Ladder, 10 to 180 kDa, Thermo Fisher Scientific). One microgram of purified recombinant cytolysins was separated on a 12% gel.

Recombinant PLY generated previously [27] was used for mouse immunisation and production of monoclonal antibodies (MAbs). In total, 10 hybridomas-secreting antibodies of different IgG subtypes were generated: IgG1 (clones 3A9, 3C10, 3F3, 9F2, 12D10, 12F11 and 14E4), IgG2a (clones 6E5 and 19G1) and IgG2b (clone 6E3) (Table 5). The specificity of the antibodies against PLY was confirmed by ELISA and Western blot (WB) (Table 5, Figure 1). The MAbs did not react with irrelevant bacterial proteins extract from *E. coli* in WB used as a negative control (data not shown). For more detailed characterisation of the interaction between the MAbs and PLY, the apparent dissociation constants (K_d_) describing the binding affinity of the MAb to the antigen were determined by an indirect ELISA. The obtained K_d_ values of the MAbs with PLY ranged from 0.2 to 5.3 nM (Table 5). The K_d_ values of the MAbs with other cytolysins also were determined, as described below.

To characterise the reactivity of the MAbs more precisely, the cross-reactivity of each MAb with a panel of recombinant cytolysins ILY, VLY, PFO, LLO, INY and SLO was tested. The ELISA and WB results showed that six MAbs (clones 3C10, 3F3, 9F2, 12D10, 12F11 and 14E4) out of 10 recognised the PLY exclusively and show no cross-reactivity with other cytolysins (Table 5). In WB, the PLY-specific MAbs revealed a protein band corresponding to 55 kDa (Figure 1c, lane 2). A different cross-reactivity pattern with four MAbs was observed. The MAb 6E5 was broadly cross-reactive and recognised five cytolysins: ILY, VLY, PFO, LLO, INY (Table 5, Figure 1d lanes 2–7). The MAb 3A9 cross-reacted with PFO and LLO (Table 5, Figure 1b, lanes 1,5,6). The cross-reactivity of MAbs 6E3 and 19G1 with ILY and VLY was observed only by ELISA however, no reactivity by WB was determined, indicating conformation-dependent epitopes (Table 5, Figure 1c, lane 2).

**Figure 2:**
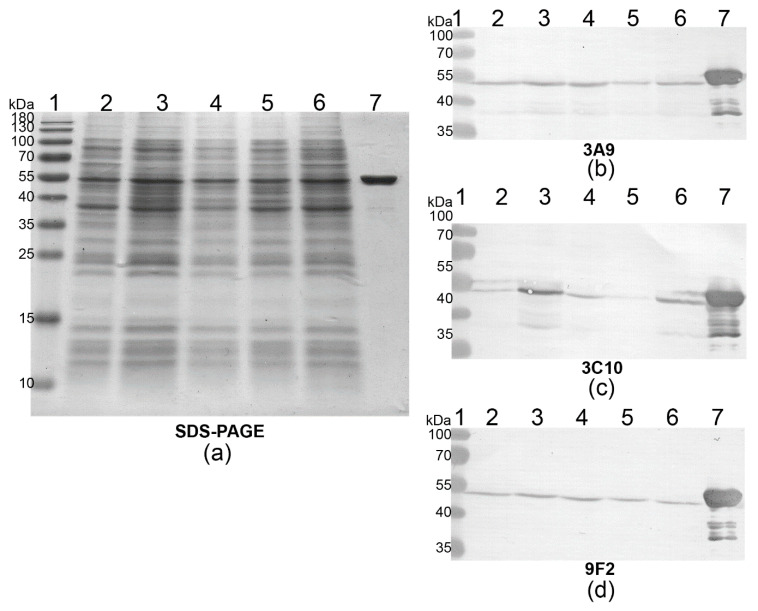
The reactivity of the MAbs with wild-type PLY from cultured *S. pneumoniae* clinical isolates 6477 (lane 2), 6840 (lane 3), 6958 (lane 4), 7123 (lane 5), 7144 (lane 6) and with 2 µg of purified recombinant PLY (lane 7). (**a**) SDS-PAGE. (**b**) WB with MAb 3A9. (**c**) WB with MAb 3C10. (**d**) WB with MAb 9F2. Lane 1, prestained molecular weight marker (PageRuler™ Prestained Protein Ladder, 10 to 180 kDa, Thermo Fisher Scientific).

The reactivity of the MAbs with wild-type PLY was investigated using cultured clinical *S. pneumoniae* isolates. For this purpose, the cell lysates of isolates 6477 (11A serotype), 6840 (14 serotype), 6958 (15B serotype), 7123 (3 serotype) and 7144 (unknown serotype) were fractionated in SDS-PAGE, and their reactivity with the MAbs was investigated by WB (Figure 2). The MAbs 3A9, 3F3, 6E5, 9F2, 12F11, 14E4, 19G1 recognised native PLY in all clinical samples, whereas the MAbs 3C10 and 12D10 reacted weakly with native PLY from isolates 6477 and 7123. A similar reactivity pattern of the MAbs 3C10 and 12D10 was observed as they recognise the same aa 403–423 PLY region (see below).

### 3.2. Mapping of MAb Epitopes

**Figure 3:**
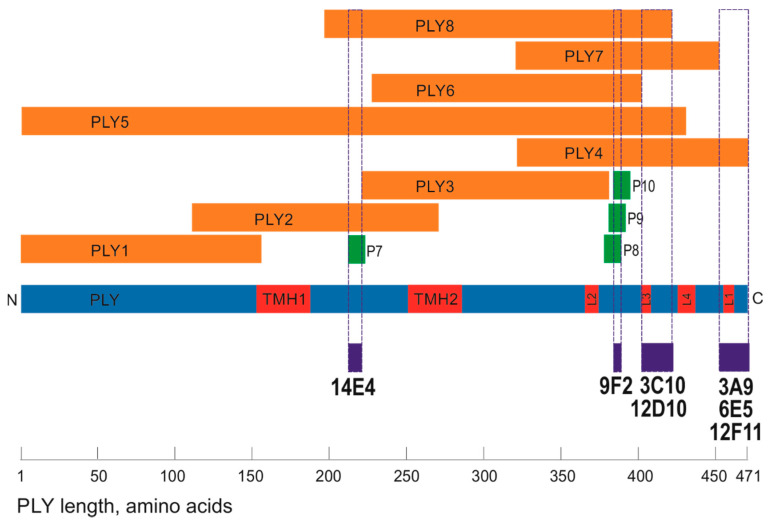
Schematic presentation of the MAb recognition sites within the overlapping fragments and peptides spanning the PLY sequence (underlined and coloured in dark blue). The full-length PLY is coloured in blue. Structural elements-transmembrane hairpins (TMH) in D3 and loops (L1–L4) in D4 are coloured in red. PLY1–PLY8, PLY fragments expressed in *E. coli* are coloured in orange. Synthetic peptides P7–P10 are coloured in green.

To identify the MAbs interaction site (epitopes) within the PLY molecule 8 overlapping PLY fragments expressed in *E. coli* (Table 1) and 25 commercial peptides spanning the respective PLY sequences (Table 3) were used. The epitope mapping scheme is presented in Figure 3. First, the reactivity of the MAbs with each recombinant His-tagged PLY fragment was investigated. The biosynthesis of the recombinant PLY fragments in *E. coli* cells was confirmed by SDS-PAGE of cell lysates under denaturing and reducing conditions and by WB with anti-His-Tag MAb (data not shown). WB revealed the epitopes of seven out of 10 MAbs (Table 6). The MAb 14E4 recognised aa 196–220 within PLY molecule as it reacted with fragments 2, 5, 8. The MAb 9F2 reacted with fragments 4–8, suggesting its binding site within aa 381–402 of PLY. The MAbs 3C10 and 12D10 recognised overlapping sequence of fragments 4, 5, 7 and 8, comprising PLY sequence of aa 403–423. MAbs 3A9, 6E5 and 12F11 recognized regions comprising aa 454–471. The epitopes of the MAbs 9F2, 3C10, 12D10, 3A9, 6E5 and 12F11 were localised in D4 of PLY (aa 362–471) [40]. Based on the available PLY structures, the epitopes recognised by the MAbs considering the tertiary structure of PLY (5AOD), were localised in D1 and in the L1 and L3 loops at the tip of D4.

**Figure 4:**
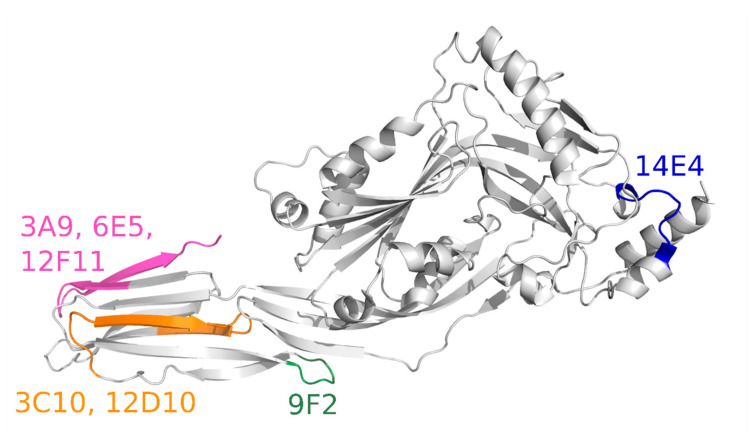
The regions of PLY necessary for recognition of MAb 14E4 (aa 212–220, coloured in blue), 9F2 (aa 384–389, coloured in green), MAbs 3C10, 12D10 (aa 403–423, coloured in orange), MAbs 3A9, 6E5, 12F11 (aa 454–471, coloured in pink) on PDB structure 5AOD.

For fine epitope mapping, the reactivity of the MAbs with overlapping synthetic peptides spanning the identified recognition sites aa 196–220, aa 381–423 and aa 454–471 of PLY was measured by an indirect ELISA. Peptide sequences and the identified MAb recognition sites are presented in Figure 3. The MAb 14E4 was reactive with only one peptide P7, which allows the exact epitope localisation: the 9 aa-long sequence DVFQDTVTV between aa 212 and 220 of PLY (Figure 3, Figure 4, coloured in blue). The epitope YDHQGK recognised by the MAb 9F2 was identified based on its reactivity with three overlapping peptides (P8–P10) spanning PLY sequence between aa 384 and 389 (Figure 3, Figure 4, coloured in green). The attempts to localise the exact epitopes of the MAbs 3C10, 12D10, 3A9, 6E5, 12F11, 3F3, 6E3 and 19G1 were unsuccessful as they did not interact with any of the synthetic peptides.

### 3.3. Identification of PLY-Neutralising MAbs and Determination of Their Neutralisation Potency

**Figure 5:**
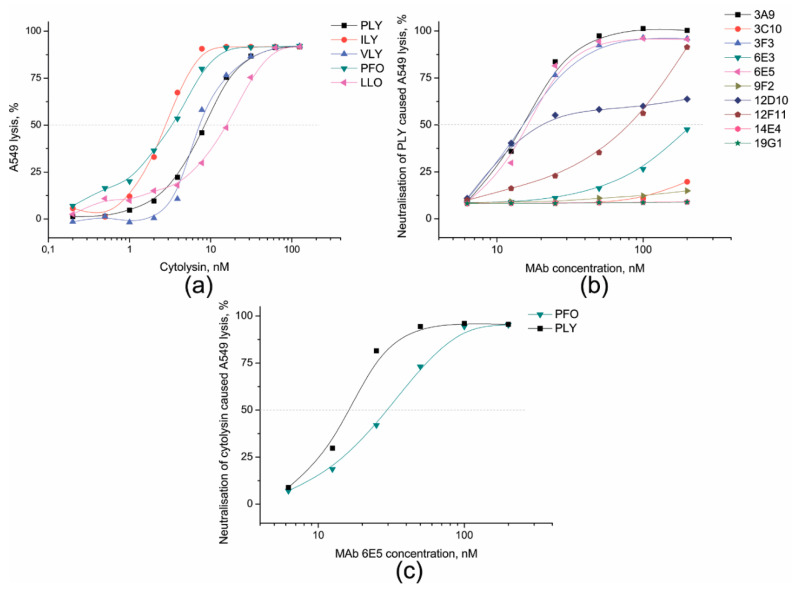
The dose-dependent curves representing cytolysis of human lung epithelial A549 cells induced by PLY, ILY, VLY, PFO, and LLO (**a**), neutralisation of 1HU PLY induced cytolysis by the MAbs 3A9, 3C10, 3F3, 6E3, 6E5, 9F2, 12D10, 12D11, 14E4, 19G1 in range of centration 6.3–200 nM (**b**) and neutralisation of 1HU of PFO-induced lysis by the MAb 6E5 within a concentration range of 6.3–200 nM (**c**).

It is known that many human cell types are susceptible to cell lysis induced by PLY or other bacterial PFT [41]. Therefore, we investigated the susceptibility of human lung epithelial cell line A549 to PLY-induced cytolysis and the ability of the MAbs to neutralise PLY-induced cytolysis. In the first step, the dose-dependent cytolytic activity of PLY was investigated at concentrations ranging from 250 to 0.2 nM (Figure 5a). The 50% cytolysis of A549 cells was detected at 7.2 nM PLY (Figure 5a, black curve with squares). This PLY concentration was set as one hemolytic unit (HU) and used for testing the neutralising activity of the MAbs. For the neutralisation test, PLY was preincubated with serial dilutions of the MAbs within a concentration range of 6.25–200 nM. The neutralisation unit (NU) for each MAb was calculated from the MAb concentration-dependent curves and was set as a required MAb concentration to reduce PLY cytolytic activity by 50%. Lower NU values indicate higher neutralising potency of the MAbs. For MAbs 3A9, 3F3, and 6E5, their concentrations ranging from 78 to 83 nM were required to neutralise 1 HU (7.2 nM) of PLY, whereas the NU of the most potent neutralising MAbs 3A9, 3F3, 6E5 and 12D10 ranged between 15.1 and 20.6 nM (Figure 5b). The NU of the MAb 12F11 was 79.4 nM. Thus, the latter antibody was categorised as weakly neutralising (Figure 5b).

The cross-reactive MAb 6E5 was able to inhibit PFO-mediated A549 cells lysis. Twenty-nine nM of the MAb 6E5 was sufficient to neutralise 50% of PFO 1 HU-induced cyolysis (Figure 5c.). To prove whether this MAb is able to neutralise other cytolysins, the additional cross-neutralisation experiments were performed with ILY, VLY and LLO, but no inhibition activity was observed (data not shown).

### 3.4. Investigation of MAb Interference with the PLY-Cholesterol Binding

**Figure 6:**
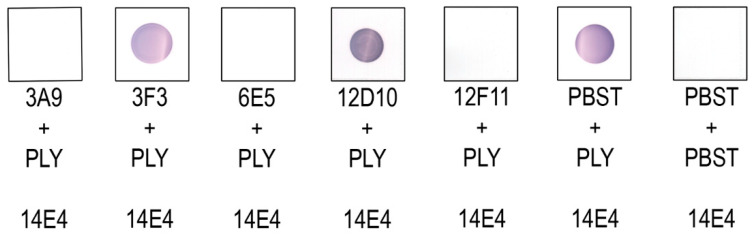
Inhibition of PLY binding to the immobilised cholesterol (1 µM/spot) by the neutralising MAbs 3A9, 3F3, 6E5, 12D10, 12F11 performed by the cholesterol dot blot assay.

According to epitope mapping data, the neutralising MAbs are directed against the L1 (MAbs 3A9, 6E5 and 12F11) and L3 (MAb 12D10) loops within the D4 (Figure 4). The D4 is crucial for PFT interaction with cholesterol-rich membranes [14,18]. To prove the hypothesis that the neutralising MAbs interfere with cholesterol-binding site located in the L1 loop, the dot blot assay with the immobilised cholesterol was performed. The neutralising MAbs 3A9, 6E5, 12F11, 12D10 and 3F3 were preincubated with PLY at molar ratio 2: 1 and loaded for a reaction with 1 µM of cholesterol spotted on the nitrocellulose membrane. The MAb 14E4 recognising aa 212–220 of PLY D1 (not involved in membrane binding) was used for the detection of the cholesterol-PLY complex on the membrane. The MAbs 3A9, 6E5, 12F11 showed a similar recognition pattern as they blocked PLY binding to the immobilised cholesterol (Figure 6). In contrast, the MAbs 3F3 and 12D10 did not interfere with the PLY-cholesterol binding as concluded from their reactivity with PLY bound to the immobilised cholesterol (Figure 6).

### 3.5. Investigation of MAb Interference with the PLY-MRC-1 Binding

Recent data suggest that PLY at sublytic doses can activate complement [21], rearrange the cytoskeleton of host cells [23], and inhibit proinflammatory cytokine responses [22]. The receptor of PLY promoting anti-inflammatory responses and enhancing *S. pneumoniae* survival in human cells is mannose receptor C type 1 (MRC-1) [19]. Therefore, we evaluated the ability of the MAbs to inhibit the interaction of PLY with MRC-1 in vitro. For this test, we performed an indirect ELISA with the recombinant MRC-1 immobilised in the 96-well plate. Individual MAbs at concentrations ranging from 3 to 200 nM were preincubated with PLY (72 nM) and added to the plate immobilised with MRC-1. Two MAbs 3F3 and 6E5 showing high affinity to PLY (Table 5) inhibited the PLY-MRC-1 interaction at a concentration of 200–50 nM (Figure S1). In contrast, the MAbs 3A9, 3C10, 6E3, 9F2, 12D10, 12F11, 14E4 and 19G1 did not inhibit PLY-MRC-1 interaction even at 200 nM concentration (Figure S1). The binding efficiency of the MAb-PLY complex to MRC-1 was lower but consistent with MAb reactivity to PLY (Table 5). According to the MAb 6E5 epitope identified within aa 454–471, PLY binding to MRC-1 site is localised in L1 of D4. The region involved in PLY interaction with MRC-1 was confirmed by testing synthetic peptides spanning PLY sequence within aa 454–471 binding to recombinant MRC-1 immobilised in the ELISA plate. The peptide P21 corresponding to aa 450-KRTISIWGTTL-460 of PLY was identified as strongly reactive with MRC-1 (Figure S2, black curve with squares). The peptide P22 corresponding to aa 453-ISIWGTTLYPQ-463 of PLY was identified as weakly reactive with MRC-1 (Figure S2, red curve with circles). Both peptides contain the aa residue (W456) recognised by the computational analysis (see below) as necessary for the interaction of the highly neutralising MAb 6E5 with PLY.

### 3.6. Computational Analysis of PLY Regions Recognised by the Neutralising MAbs

**Figure 7:**
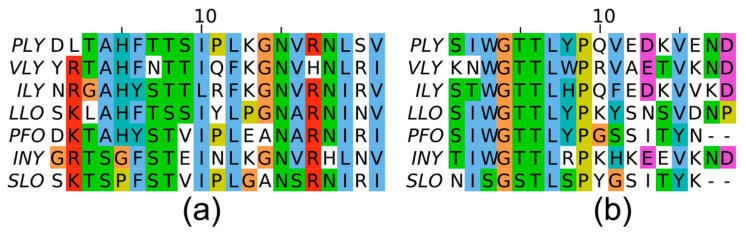
Sequence alignment of cytolysins (PLY, ILY, VLY, PFO, LLO, INY and SLO) representing binding sites for the neutralising MAbs: aa 403–423 recognition site of the MAb 12D10 (**a**), aa 454–471 recognition site of the MAbs 3A9, 6E5, 12F11 (**b**).

Our attempts to identify the exact PLY epitopes recognised by the neutralising MAbs 3A9, 6E5, 12F11 and 12D10 were unsuccessful as these MAbs did not interact with the overlapping synthetic peptides. To solve this issue, we employed in silico approaches to predict these epitopes localised within the loops L1 and L3. The sequences of cytolysins PLY, ILY, VLY, PFO, LLO, INY and SLO were aligned using the multiple sequence alignment program MAFFT. The alignments of aa residues representing recognition sites of the MAbs (aa 454–471 site for 3A9, 6E5, 12F11, aa 403–423 for 12D10) are shown in Figure 7. The MAb 6E5 was cross-reactive with PLY, ILY, VLY, PFO, LLO, INY, but did not react with SLO (Table 5, Figure 1), the MAb 3A9 cross-reacted with PLY, PFO and LLO, whereas the MAbs 12F11, 12D10 were exclusively specific to PLY (Table 5). Sequence alignments were analysed and compared to the MAb cross-reactivity pattern, allowing epitope prediction (Figure 7).

In the next step, we analysed the spatial patterns of cytolysins recognised by the MAbs. Protein structures and their solvent-accessible surfaces were compared between the available structures of cytolysins using contact area differences (CAD-score). Local CAD-score values were used to compare the solvent-accessible regions of 21 aa (aa 403–423 of PLY) and 18 aa (aa 454–471 of PLY) binding sites of each cytolysin to PLY (Tables S1 and S2 respectively).

**Figure 8:**
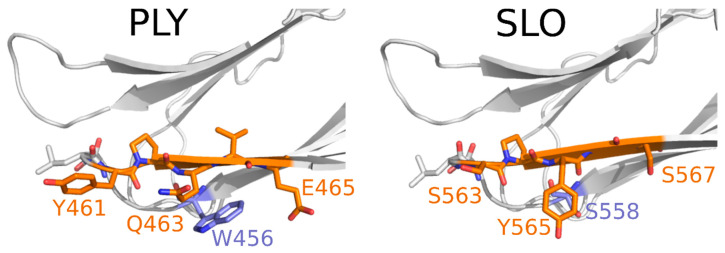
MAb binding sites in D4 of PLY (**left**) and SLO (**right**). Tryptophan W456 (PLY) and serine S558 (SLO) are shown in violet; the other predicted epitope residues are coloured in orange.

When analysing the PLY surfaces recognised by the broadly cross-reactive MAb 6E5, we identified the differences among PLY, VLY, ILY, LLO, PFO, INY and SLO. Although the structure of cytolysins and their surfaces were highly similar to each other (CAD-score ≥ 0.7, see Tables S1 and S2), there were remarkable differences in the surface of the C-terminal part of SLO, which may account for differences in antibody binding. First, tryptophan at position 456 (W456) in PLY and the respective aa residue in ILY, VLY, PFO, LLO, INY is changed to serine (S558) in SLO (Figure 7). It is known that tryptophan is frequently a “hot spot” aa residue in protein-protein binding sites [42,43,44]. Therefore, it is not surprising that substitution of this large residue to a smaller and hydrophilic serine abolishes binding of SLO to the MAb. Remarkable differences were also observed in the PLY region spanning the residues 461-YPQVE-465. These may account for differences in binding affinity between PLY, LLO, PFO and SLO. For instance, several PLY aa residues are different compared to SLO: tyrosine Y461 is changed to serine (S563) in SLO (large aromatic aa to a small aliphatic aa residue), glutamine Q463 is changed to tyrosine (Y565) in SLO (aliphatic to aromatic aa residue), glutamic acid E465 is changed to serine (S567) in SLO (negative to uncharged aa residue) (Figure 8). This also confirms the assumption that the epitope of the MAb 6E5 is a conformation-dependent (no binding to synthetic peptides), as aa residues 461–465 are in close proximity to tryptophan W456 that is considered a necessary aa residue for MAb binding.

The differences in binding specificity of MAbs 3A9 and 12F11 for different cytolysins are harder to explain, however the same residues may also be important for the interaction in the region of aa 454–471. The comparison of binding sites recognized by the MAb 12D10 (within PLY region aa 403–423) did not show any clear differences. A more in-depth computational analysis combined with experimental methods might be required for further investigation of this region.

## 4. Discussion

PLY is known as an important protein virulence factor expressed by most disease-causing isolates of *S. pneumoniae* [45,46]. The PLY-specific MAbs may represent valuable reagents for a better understanding of the role of PLY in pneumococci pathogenesis or neutralisation of PLY activity. To our best knowledge, just one collection of the well-characterised MAbs against the native PLY was described previously [9,28,29,47,48]. De Los Toyos and colleagues (1996) generated a collection of nine MAbs against PLY. The MAbs were used in functional and structural studies of PLY [28,29,48], for developing the quantitative PLY tests [47], and protection against pneumococcal pneumonia in mice [9]. Six out of nine MAbs showed the ability to neutralise PLY-induced erythrocyte lysis [28].

In this study, we have generated ten hybridomas producing high-affinity MAbs against recombinant PLY. *E. coli*-expressed His-tagged PLY was used for the immunisation of mice [27,32]. The ability of the MAbs to recognise wild-type PLY from *S. pneumoniae* clinical isolates was demonstrated, thus confirming the antigenic similarity of recombinant and native PLY. The regions recognised by the newly generated MAbs were determined using a series of overlapping truncated PLY variants and synthetic peptides. The MAbs 3F3, 6E3 and 19G1 did not show any reactivity with PLY fragments as well as overlapping peptides, suggesting conformation-dependent binding and interaction with aa residues from different spatial patterns of PLY. One MAb 14E4 was found to recognise PLY epitope in D1 comprising aa 212-DVFQDTVTV-220 (5AOD, Figure 4). No relevant data about the functionality of this PLY sequence was found. Therefore, the MAb 14E4 might be used for PLY-specific detection as a universal tool that does not interfere with PLY functions. Six out of 10 MAbs (clones 3A9, 3C10, 6E5, 9F2, 12D10, 12F11) recognise PLY epitopes in D4. The position of the epitopes within D4 is consistent with that of recognised by previously described MAbs against PLY and PFO [28,49,50]. The previous characterisation of MAbs PLY-4, PLY-5, and PLY-7 by De Los Toyos and colleagues determined the epitope of PLY-7 to be aa 401-GQDLTAH-407 [28]. The epitope of PLY-7 recognised sequence aa 401-GQDLTAH-407. In addition, the suilysin (SLY) epitope 429-GVNLTSH-435 was recognised by this MAb [48]. PLY-5 directed to the extreme C-terminal part of PLY was shown to cross-react with SLO, LLO, suilysin, cereolysin O, tetanolysin, alveolysin, thuringiolysin O, ivanolysin and seeligerolysin. It recognises the epitope 433-WEWWRT-438 within the conserved tryptophan-rich motif [29] responsible for conformational changes during monomer-monomer interaction of cytolysins and anchoring into membrane bilayer [13,16,51].

In contrast to the MAbs raised against the native PLY, previous attempts to generate PLY-neutralising MAbs against UDP using the synthetic peptide coupled to bovine serum albumin (BSA) as an immunogen were unsuccessful. Seven anti-peptide MAbs were broadly cross-reactive with other cytolysins, but were non-neutralising, most probably due to their low-affinity binding to cytolysins [25].

In our study, the strategy of fine epitope mapping based on a series of synthetic overlapping peptides was not successful for the neutralising MAbs 3A9, 3C10, 6E5, 12D10, 12F11 as they did not interact with any of the peptides. The similar reactivity pattern was observed for the previously described neutralising MAbs: PLY-4 against PLY [28] and the MAb 9B4 against VLY [27]. The PLY epitope recognised by the MAb PLY-4 was conformation-dependent and has a core reactivity with PLY aa residue R232 [48].

The variability of the ply gene is about 3.3% [46]. This might have an impact on the PLY-MAb interaction. An example of the MAb PLY-7 recognising aa 401–407 in PLY shows that not every MAb is suitable for PLY detection in clinical samples as it might give false-negative results [46] due to the most prevalent mutation at PLY aa 402 substituting Q to E [50]. In line with these observations, we found that two MAbs 3C10 and 12D10 recognising aa 403–423 sequence within PLY L3 (5AOD, Figure 4) interacted weakly with PLY from two clinical cultures of *S. pneumoniae*. This suggests that these MAbs are not recommended for PLY detection in biological samples. These MAbs might be suitable for studying the prevalence of *S. pneumoniae* isolates having the PLY mutation at aa 402.

It is known that PLY is involved in activation of the complement system through binding to the Fc region of immunoglobulin G (IgG) [11,20,21]. Thetyrosine Y384 and aspartic acid D385 of PLY located at D4 are responsible for this interaction [20]. PLY mutations around these aa residues eliminate complement activation [11]. We have generated the MAb 9F2 recognising the epitope 384-YDHQGK-389 within PLY region involved in the complement activation process. This MAb does not neutralise the cytolytic activity of PLY, but it might represent a potential tool for blocking the PLY–complement interaction. This presumption is not yet proven and requires further experiments.

The binding sites of the newly developed MAbs 3A9, 6E5 and 12F11 were located within L1 of D4 (5AOD, Figure 4). The MAbs 3A9, 6E5, 12F11, 12D10 were able to inhibit PLY-induced cytolysis of human lung epithelial A549 cells. For a better understanding of the epitopes recognised by these MAbs, we analysed the surface-exposed aa residues within the D4 of PLY and related cytolysins (VLY, ILY, LLO, PFO, INY, SLO) by computational tools. The alignment of aa 454–471 region in different cytolysins revealed that tryptophan W456 in PLY, VLY, ILY, LLO, PFO, INY is changed to serine (S558) in SLO. The substitution of tryptophan W456 to serine (S558) in SLO is the most remarkable for the cross-reactive MAb 6E5 recognition of highly diverse but surface-similar epitope of VLY, ILY, LLO, PFO, INY. The tyrosine Y461 (changed to serine (S563) in SLO), glutamine Q463 (changed to tyrosine (Y565) in SLO) and glutamic acid E465 (changed to serine (S567) in SLO) were also suggested as highly significant for the interaction of the MAb 6E5 with VLY, ILY, LLO, PFO, INY.

We have generated MAbs 3A9, 6E5, 12F11, 12D10, 3F3 capable of neutralising PLY-induced cytolysis. The interaction of PLY with immobilised cholesterol revealed that the MAbs 3A9, 6E5 and 12F11 inhibit PLY binding to cholesterol by covering the L1 responsible for recognition of cholesterol on cell membranes [18]. The MAb 12D10 recognises aa 403–423 including the L3 within the D4. The recognised sequence is close to the UDP (L4) promoting monomer–monomer interactions and pore formation [13]. These findings suggest that the MAb 12D10 binding site probably overlaps with the UDP thus preventing the oligomerisation of PLY and forming the pore.

Besides PLY cytolytic activity, we have analysed the ability of the newly generated MAbs to neutralise other pathogenic effects of PLY. It is known that PLY has several ways to act on host cells. At sublytic doses, PLY interacts with the cellular receptor MRC-1 in human cells. By this way, pneumococci invade alveolar macrophages and become resistant against cell immune system factors [19]. In this study, we identified two MAbs (clones 3F3 and 6E5) capable of inhibiting the PLY binding to MRC-1 in vitro, most probably by covering the PLY-MRC-1 interaction site. Based on the MAb 6E5 epitope mapping data and its ability to inhibit PLY binding to MRC-1, the PLY region involved in its interaction with MRC-1 is located within the D4 at aa 454–471 of PLY. This assumption was verified by MRC-1 interaction with synthetic peptides spanning aa 454–471 sequence of PLY.

Summarising the cross-reactivity and neutralisation data, the broadly cross-reactive neutralising MAb 6E5 may represent a promising tool for the reduction of the pathogenic effects of PLY and related CDCs.

## 5. Conclusions

A new collection of MAbs raised against recombinant PLY is described. By combining experimental and computational approaches, the linear and conformation-dependent epitopes of the MAbs have been investigated in detail. The capability of some MAbs to neutralise PLY pathogenic effects, such as cytolytic activity and binding to the cellular receptor MRC-1, has been demonstrated. Five MAbs recognising the epitopes within PLY D4 have been shown to neutralise PLY cytolytic activity. Three of them inhibited PLY binding to cholesterol. Two MAbs inhibiting the interaction of PLY with its cellular receptor MRC-1 have been identified, which allowed the mapping of the PLY binding site to MRC-1. A unique neutralising MAb (clone 6E5) directed to the cholesterol-binding loop of PLY, blocking the PLY interaction with its cellular receptor MRC-1 and showing a broad cross-reactivity with several CDCs has been identified. The newly developed MAbs represent a promising tool for studying PLY or similar CDCs.

## Figures and Tables

**Table 1:** The list of truncated pneumolysin (PLY) fragments: their DNA (bp) and amino acids (aa) sequences.

Truncated PLY Fragment	DNA Sequence, bp	Protein Sequence, aa
PLY1	1–468	1–156
PLY2	331–810	111–270
PLY3	661–1140	221–380
PLY4	961–1413	321–471
PLY5	1–1293	1–431
PLY6	676–1206	226–402
PLY7	961–1359	321–453
PLY8	586–1269	196–423
Full-length PLY (accession number: WP_001284359)	1–1416	1–471

**Table 2:** The primers used to amplify the truncated PLY fragments.

Primer	Primer Sequence
PLY1_F	5′-GAGCATATGTTAGTGAGCCGTGATTTTT-3′
PLY1_5_R	5′-GCCCATATGATGGCAAATAAAGCAGTAA-3′
PLY2_F	5′-GGCCATATGTTAGACTCCTTTTATCAAAGC-3′
PLY2_R	5′-GATCATATGAGCTTTCTCCAAGTGGAAGAC-3′
PLY3_F	5′-GGCCATATGTTAATCCCAAGTAATATAATA-3′
PLY3_R	5′-ATTCATATGGAGGATTTAAAACAGAGAGGA-3′
PLY4_F	5′-GCGCATATGTTAGTCATTTTCTACCTTATC-3′
PLY4_R/PLY_7R	5′-ATACATATGACAGCAGATCATCCAGGCTTG-3′
PLY5_F	5′-ATTCATATGTTAAAGCCCGGTACACTCTCT-3′
PLY6_F	5′-ATAGGATCCTTACTGCCCATTTCTGTCCCA-3′
PLY6_R	5′-ATCCATATGAGAGGAATTTCTGCAGAGCGT-3′
PLY7_F	5′-ATAGGATCCTTAATTCGTCCGCTTACGCAC-3′
PLY8_F	5′-ATAGGATCCTTAGACAGAGAGATTACGAAC-3′
PLY8_R	5′-ACGCATATGAAGCAGATTTATTATACAGTC-3′

**Table 3:** Synthetic peptides used for epitope mapping.

I recognition site within PLY	196-KQIYYTVSVDAVKNPGDVFQDTVTV-220
P1	194-NFKQIYYTVSVD-205
P2	197-QIYYTVSVDAVK-208
P3	200-YTVSVDAVKNPG-211
P4	203-SVDAVKNPGDVF-214
P5	206-AVKNPGDVFQDT-217
P6	209-NPGDVFQDTVTV-220
P7	212-DVFQDTVTVEDL-223
II recognition site within PLY	382-LSYDHQGKEVLTPKAWDRNGQDLTAHFTTSIPLKGNVRNLSV-423
P8	378-TWDELSYDHQGK-389
P9	381-ELSYDHQGKEVL-392
P10	384-YDHQGKEVLTPK-395
P11	387-QGKEVLTPKAWD-398
P12	390-EVLTPKAWDRNG-401
P13	393-TPKAWDRNGQDL-404
P14	396-AWDRNGQDLTAH-407
P15	399-RNGQDLTAHFTT-410
P16	402-QDLTAHFTTSIP-413
P17	405-TAHFTTSIPLKG-416
P18	408-FTTSIPLKGNVR-419
P19	411-SIPLKGNVRNLS-422
P20	414-LKGNVRNLSVKI-425
III recognition site within PLY	454-SIWGTTLYPQVEDKVEND-471
P21	450-KRTISIWGTTL-460
P22	453-ISIWGTTLYPQ-463
P23	456-WGTTLYPQVED-466
P24	459-TTLYPQVEDKVE-469
P25	462-YPQVEDKVEND-471

Amino acids assigned to the MAbs recognition sites are underlined. The peptides were synthesised with N-terminal biotin. For a better spatial presentation of the target peptide, a linker sequence-SFGSG-was inserted between biotin and peptide.

**Table 4:** The structures of cytolysins used for domain 4 (D4) surface comparison.

Toxin	Structure	D4 Start
PLY	5AOD	361
VLY	5IMY *	406
ILY	1S1R	419
LLO	4CDB *	417
PFO	1PFO	392
INY	-	-
SLO	4HSC	463

* A structure of cytolysin with 98% aa sequence identity with the target protein D4 (two substituted residues).

**Table 5:** Characterisation of the monoclonal antibodies (MAbs) raised against recombinant PLY.

MAb Clone No.	IgG Subtype	Assay	MAb Reactivity and Cross-Reactivity with Cytolysins						
			PLY	ILY	VLY	PFO	LLO	INY	SLO
6E5	IgG2a	ELISA ^1^	0.6	0.2	0.4	1.7	67.0	0.8	-
		WB ^2^	+	+	+	+	+	+	-
3A9	IgG1	ELISA	0.2	-	-	0.5	67.0	-	-
		WB	+	-	-	+	+	-	-
3F3	IgG1	ELISA	4.8	-	-	-	-	-	-
		WB	+	-	-	-	-	-	-
12D10	IgG1	ELISA	0.6	-	-	-	-	-	-
		WB	+	-	-	-	-	-	-
12F11	IgG1	ELISA	0.5	-	-	-	-	-	-
		WB	+	-	-	-	-	-	-
6E3	IgG2b	ELISA	1.7	5.5	4.1	-	-	-	-
		WB	+	-	-	-	-	-	-
19G1	IgG2a	ELISA	5.3	3.8	3.7	-	-	-	-
		WB	+	-	-	-	-	-	-
3C10	IgG1	ELISA	0.5	-	-	-	-	-	-
		WB	+	-	-	-	-	-	-
9F2	IgG1	ELISA	0.2	-	-	-	-	-	-
		WB	+	-	-	-	-	-	-
14E4	IgG1	ELISA	0.3	-	-	-	-	-	-
		WB	+	-	-	-	-	-	-

^1^ ELISA results are presented as dissociation constant (K_d_) values (nM) obtained from ELISA titration curves. The K_d_ is defined as the MAb concentration (nM) that gives 50% of the maximum binding with the respective antigen; ^2^ WB—a visible band defined as a positive reaction “+”, no band defined as a negative reaction “−”.

**Table 6:** The results of MAb epitope mapping using a series of overlapping PLY fragments.

MAb Clone	Identified MAb Recognition Sites, aa	Protein Structure Elements	PLY Fragments							
			1	2	3	4	5	6	7	8
14E4	196–220	D1		+			+			+
9F2	381–402	D4				+	+	+	+	+
3C10	403–423	L3 in D4				+	+		+	+
12D10						+	+		+	+
3A9	454–471	L1 in D4				+				
6E5						+				
12F11						+				
3F3	not determined						+			
6E3							+			
19G1							+			

“+” positive reaction in WB.

## Supplementary Materials

The following are available online at https://www.mdpi.com/2218-273X/10/7/1009/s1, **Figure S1:** The inhibition of PLY-MRC-1 interaction by the MAbs 3A9, 3C10, 3F3, 6E3, 6E5, 9F2, 12D10, 12D11, 14E4, 19G1. The test was performed by an indirect ELISA using immobilised recombinant MRC-1. The figure shows the reactivity (OD) of individual MAb (200 nM) preincubated with PLY (72 nM). **Table S1:** CAD-score values showing similarities of protein structures and surfaces (1: identical structure. 0: no similarity) in PLY aa 403–423 region. Substantial differences and potentially significant residues are marked in bold. **Table S2.** CAD-score values showing similarities of protein structures and surfaces (1: identical structure. 0: no similarity) in PLY aa 454–471 region. Substantial differences and potentially significant residues are marked in bold.
