# Supplementary material for: Mapping of Recognition Sites of Monoclonal Antibodies Responsible for the Inhibition of Pneumolysin Functional Activity
Source: Biomolecules. 2020 Jul 8;10(7):1009. doi: 10.3390/biom10071009 (PMC7408604; doi:10.3390/biom10071009)
Supplement: Supplementary file 1 [file biomolecules-10-01009-s001.zip › Supplementary file_Tables S1.docx.pdf]

Supplementary Table S1. CAD-score values showing similarities of protein structures and surfaces (1: identical structure. 0: no similarity) in PLY aa 403-423 region. Substantial differences and potentially significant residues are marked in bold.

		**PLY**	**VLY**	**ILY**	**LLO**	**PFO**	**INY_model**	**SLO**
Binding constant 12D10		*0.6*	-	-	-	-	-	-
Global CAD-score		1.00						
Surface CAD-score		1.00						
Binding site surface score		1.00	0.69	0.65	0.72	0.79	0.76	0.72
Local residue scores								
D403	**92.43**	1.00	0.58	0.98	0.76	0.99	0.55	0.75
L404	10.92	1.00	0.00	0.00	0.00	0.00	0.90	0.00
T405	**83.53**	1.00	0.89	0.57	0.52	0.98	0.94	0.95
A406	48.23	1.00	0.44	0.77	0.93	0.27	0.96	0.99
H407	**128.54**	1.00	0.83	0.69	0.98	0.92	0.57	0.85
F408	27.72	1.00	0.44	0.28	0.83	0.01	0.10	0.60
T409	**84.39**	1.00	0.98	0.81	0.99	0.86	0.97	0.91
T410	31.01	1.00	0.87	0.88	0.82	0.97	1.00	0.35
S411	**58.31**	1.00	0.57	0.82	0.73	0.77	0.08	0.61
I412	10.02	1.00	0.84	0.56	0.80	0.52	0.48	0.56
P413	**103.98**	1.00	0.70	0.16	0.46	0.97	0.96	0.95
L414	10.02	1.00	0.63	0.46	0.96	0.81	0.60	0.77
K415	**117.37**	1.00	0.82	0.58	0.44	0.93	0.91	0.26
G416	56.19	1.00	0.76	0.70	0.71	0.21	0.88	0.24
N417	43.68	1.00	0.00	0.60	0.76	0.86	1.00	0.81
V418	1.88	1.00	0.00	0.00	0.00	0.00	0.06	0.00
R419	**93.74**	1.00	0.61	0.94	0.86	0.85	0.81	0.99
N420	64.89	1.00	0.89	0.81	0.79	0.93	0.65	0.93
L421	3.15	1.00	0.07	0.36	0.37	0.60	0.78	0.74
S422	24.69	1.00	0.00	0.00	0.49	0.00	0.72	0.00
V423	1.22	1.00	0.68	0.46	0.48	0.00	0.10	0.72

red - CAD-score <0.3

orange - CAD-score 0.31-0.5
