# Supplementary material for: Mapping of Recognition Sites of Monoclonal Antibodies Responsible for the Inhibition of Pneumolysin Functional Activity
Source: Biomolecules. 2020 Jul 8;10(7):1009. doi: 10.3390/biom10071009 (PMC7408604; doi:10.3390/biom10071009)

Supplementary Figure S1. The inhibition of the PLY-MRC-1 interaction by the MAbs 3A9, 3C10, 3F3, 6E3, 6E5, 9F2, 12D10, 12D11, 14E4, 19G1. The test was performed by an indirect ELISA using immobilised recombinant MRC-1. The figure shows the reactivity (OD) of individual MAb (200, 100, 50 nM) preincubated with PLY (72 nM).

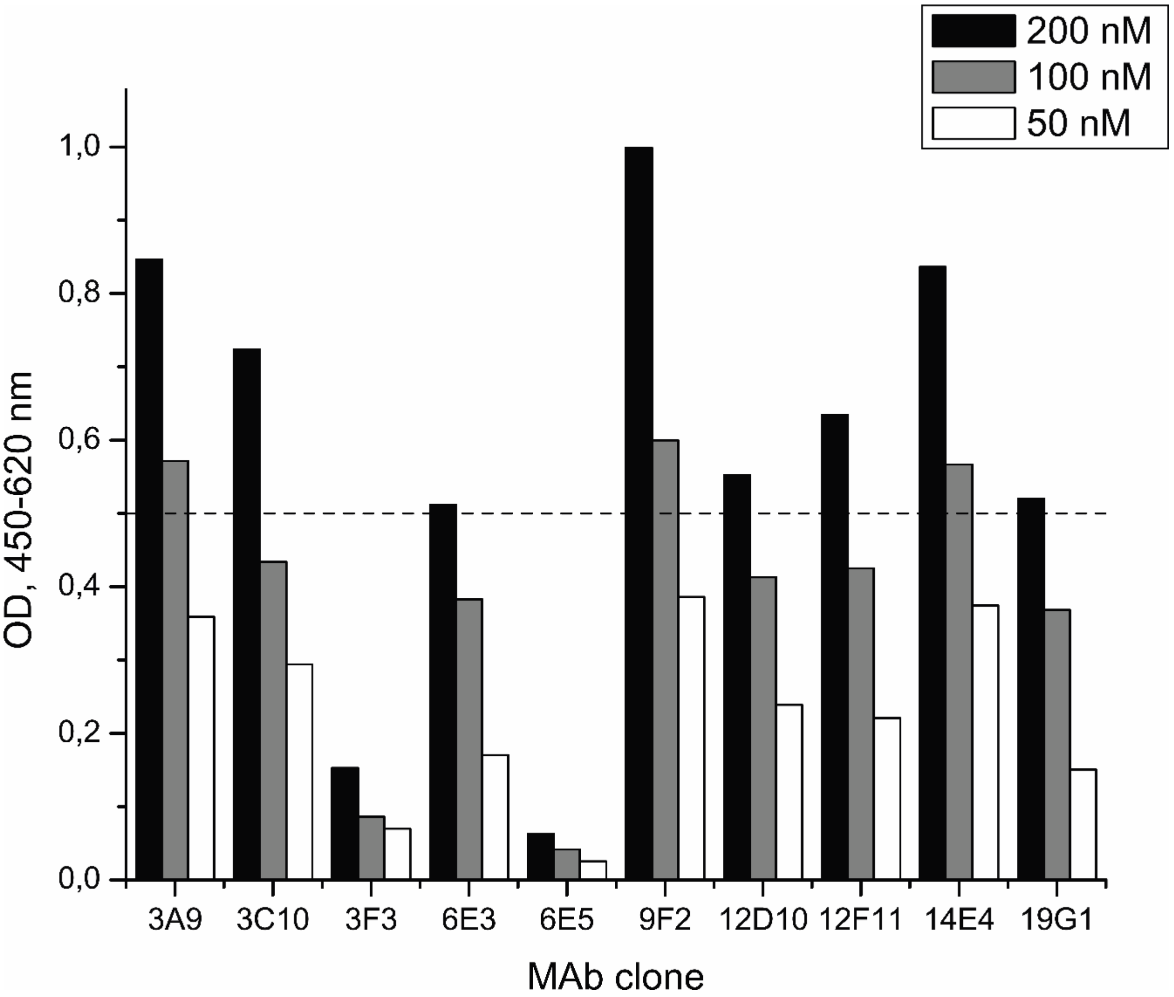

Supplement: Supplementary file 1 [file biomolecules-10-01009-s001.zip › Supplementary file_Figure S1.pdf]
