# Supplementary material for: Mapping of Recognition Sites of Monoclonal Antibodies Responsible for the Inhibition of Pneumolysin Functional Activity
Source: Biomolecules. 2020 Jul 8;10(7):1009. doi: 10.3390/biom10071009 (PMC7408604; doi:10.3390/biom10071009)
Supplement: Supplementary file 1 [file biomolecules-10-01009-s001.zip › Supplementary file_Figure S2.pdf]

**Supplementary Figure S2.** The reactivity of synthetic peptides spanning PLY sequence with recombinant MRC-1. The interaction of plate-immobilised recombinant MRC-1 with PLY peptides was detected using streptavidin-alkaline phosphatase and p-nitrophenylphosphate (pNPP) chromogen. (a) The graphic shows the reactivity (OD, optical density) of an individual peptide at 1.25-20 µg/ml concentrations with MRC-1. (b) The sequences of synthetic peptides spanning PLY respective regions.

**(a)**

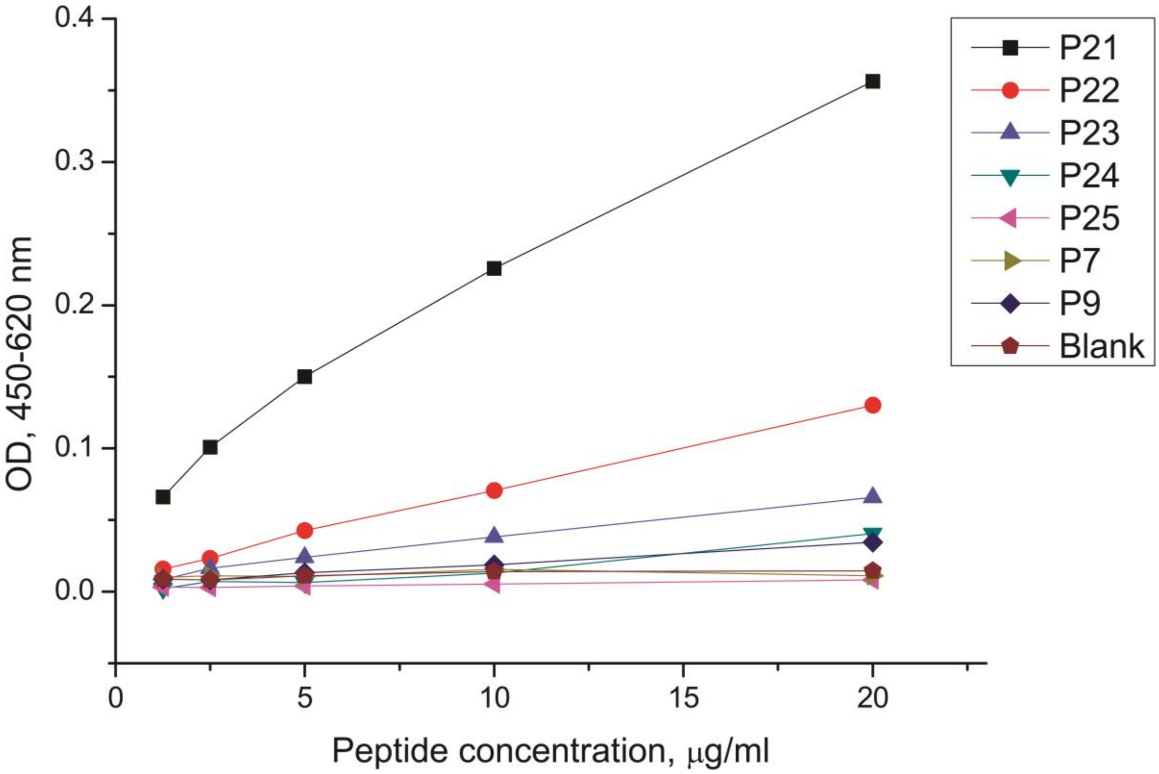


**(b)**

| | |
|---|---|
| P21 | 450-KRTISIWGTTL-460 |
| P22 | 453-ISIWGTTLYPQ-463 |
| P23 | 456-WGTTLYPQVED-466 |
| P24 | 459-TTLYPQVEDKVE-469 |
| P25 | 462-YPQVEDKVEND-471 |
| | |
| P7 | 212-DVFQDTVTVEDL-223 |
| P9 | 381-ELSYDHQGKEVL-392 |
