# Supplementary material for: Mapping of Recognition Sites of Monoclonal Antibodies Responsible for the Inhibition of Pneumolysin Functional Activity
Source: Biomolecules. 2020 Jul 8;10(7):1009. doi: 10.3390/biom10071009 (PMC7408604; doi:10.3390/biom10071009)
Supplement: Supplementary file 1 [file biomolecules-10-01009-s001.zip › Supplementary file_Table S2.docx.pdf]

**Supplementary Table S2.** CAD-score values showing similarities of protein structures and surfaces (1: identical structure. 0: no similarity) in PLY aa 454-471 region. Substantial differences and potentially significant residues are marked in bold.

| | | PLY | VLY | ILY | LLO | PFO | INY_model | SLO |
|---|---|---|---|---|---|---|---|---|
| *Binding constant 6E5* | | *0.6* | *0.4* | *0.2* | *67* | *1.7* | *0.8* | *-* |
| *Binding constant 3A9* | | *0.2* | *-* | *-* | *67* | *0.5* | *-* | *-* |
| *Binding constant 12F11* | | *0.5* | *0* | *0* | *0* | *0* | *0* | *0* |
| Global CAD-score | | 1.00 | 0.70 | 0.71 | 0.75 | 0.69 | 0.74 | 0.69 |
| Surface CAD-score | | 1.00 | 0.69 | 0.68 | 0.72 | 0.66 | 0.72 | 0.65 |
| Binding site surface score | | 1.00 | 0.67 | 0.63 | 0.68 | 0.57 | 0.73 | 0.48 |
| Local residue scores | | | | | | | | |
| S454 | 17.31 | 1.00 | 0.31 | 0.86 | 0.48 | 0.18 | 0.81 | 0.00 |
| I455 | 0.15 | 1.00 | 0.45 | 0.00 | 0.00 | 0.90 | 0.02 | 0.00 |
| **W456** | **100.90** | 1.00 | 0.88 | 0.93 | 0.99 | 0.95 | 0.86 | 0.39 |
| G457 | 21.54 | 1.00 | 0.99 | 0.78 | 0.80 | 0.98 | 0.75 | 0.90 |
| T458 | 67.51 | 1.00 | 0.81 | 0.97 | 0.93 | 0.92 | 0.69 | 0.80 |
| T459 | 38.38 | 1.00 | 0.99 | 0.29 | 0.96 | 0.59 | 0.95 | 0.96 |
| L460 | 105.32 | 1.00 | 1.00 | 0.50 | 0.97 | 0.79 | 0.52 | 0.96 |
| **Y461** | **159.34** | 1.00 | 0.93 | 0.76 | 0.96 | 0.94 | 0.92 | 0.48 |
| P462 | 15.87 | 1.00 | 0.40 | 0.00 | 0.01 | 0.00 | 0.46 | 0.85 |
| **Q463** | **85.15** | 1.00 | 0.49 | 0.92 | 0.92 | 0.20 | 0.86 | 0.14 |
| **V464** | 51.39 | 1.00 | 0.98 | 0.26 | 0.00 | 0.78 | 0.65 | 0.55 |
| **E465** | **79.99** | 1.00 | 0.53 | 0.90 | 0.26 | 0.60 | 0.30 | 0.71 |
| D466 | 57.97 | 1.00 | 0.97 | 0.85 | 0.73 | 0.66 | 0.67 | 0.48 |
| K467 | 101.27 | 1.00 | 0.73 | 0.82 | 0.79 | 0.76 | 0.93 | 0.76 |
| V468 | 74.99 | 1.00 | 0.99 | 0.93 | 0.99 | 0.88 | 0.94 | 0.87 |
| E469 | 37.25 | 1.00 | 0.00 | 0.00 | 0.00 | 0.00 | 0.00 | 0.00 |
| N470 | 108.57 | 1.00 | 0.38 | 0.47 | 0.74 | 0.00 | 0.81 | 0.00 |
| D471 | 140.60 | 1.00 | 0.00 | 0.00 | 0.00 | 0.00 | 0.67 | 0.00 |

red - CAD-score <0.3

orange - CAD-score 0.31-0.5
